# Mentalizing and Attachment From Infancy to Young Adulthood: Insights From the Metera Adoption Study

**DOI:** 10.1002/pmh.70084

**Published:** 2026-06-09

**Authors:** Pavlos Zournatzidis, Panayiota Vorria, Eirini Krana, Peter Fonagy, Patrick Luyten

**Affiliations:** ^1^ Research Department of Clinical, Educational and Health Psychology University College London London UK; ^2^ Anna Freud London UK; ^3^ Department of Psychology Aristotle University of Thessaloniki Thessaloniki Greece; ^4^ Faculty of Psychology and Educational Sciences Leuven Belgium

**Keywords:** adoption, attachment, institutional care, longitudinal, mentalizing, reflective functioning, trust

## Abstract

Mentalizing is a socio‐cognitive capacity that develops within early attachment relationships, but institutional care may disrupt this process. Although adoption significantly improves developmental outcomes, it remains unclear whether socio‐cognitive vulnerabilities observed in children persist in adulthood. This preregistered study aimed to explore (a) socio‐cognitive catch‐up in adult adoptees, and (b) predictors of reflective functioning (RF) in adulthood with a particular focus on attachment and interpersonal trust. Sixty‐eight individuals were followed from infancy to adulthood. Adoptees did not differ from nonadoptees in RF in adulthood (Cohen's *d* = 0.07, 95% CI [−0.43, 0.57]), and age at adoption was not associated with RF (*r* = 0.02, *p* = 0.917). Cross‐sectionally, RF was significantly correlated with dismissing (*r* = −0.58, *p* < 0.001) and preoccupied states of mind (*r* = 0.29, *p* = 0.017). Attachment in infancy (strange situation), preschool (attachment story completion task), and adolescence (child attachment interview) did not predict RF in adulthood. Interpersonal trust in adolescent and adult friendships was not associated with RF in adulthood. Overall, the findings suggest recovery in socio‐cognitive development among adoptees with early institutional care and indicate that early attachment may not linearly predict RF. Future research should further explore developmental pathways to better understand the precursors of mentalizing in adulthood.

## 1 Introduction

Mentalizing or reflective functioning (RF) is a socio‐cognitive capacity that pertains to the ability to recognize and interpret one's own and others' behavior in terms of mental states (Fonagy et al. 2002). As a multidimensional construct, mentalizing is closely related to other socio‐cognitive abilities (e.g., theory of mind) (Luyten et al. 2020). From a theoretical perspective, mentalizing is rooted early in life in the context of attachment relationships. In secure and sensitive caregiving, contingent and marked mirroring supports infants in recognizing and organizing their internal experiences, facilitating the development of second‐order representations of mental states (Fonagy et al. 2002; Gergely and Watson 1996).

While early attachment relationships may provide the foundation for mentalizing, recent theoretical models also emphasize the continued importance of interpersonal experiences and social learning (Fonagy et al. 2017). Central to this expanded framework is epistemic trust: the capacity to perceive information from others as personally relevant, reliable, and generalizable (Campbell et al. 2021; Fonagy and Allison 2014). Epistemic trust is thought to emerge within early attachment relationships and facilitate positive social learning, which in turn may influence the capacity to mentalize (Luyten et al. 2020).

Institutional rearing, however, is associated with elevated rates of insecure and particularly disorganized attachment (Lionetti et al. 2015) which may hinder the development of RF and negatively influence one's capacity to engage with and learn from interpersonal relationships. Although adoption is widely considered an effective intervention that can promote recovery of early developmental impairments (van IJzendoorn and Juffer 2006), catch‐up appears to be moderated by various factors such as age at adoption (a proxy of early adversity), the quality of the adoptive environment (i.e., parental mentalizing) as well as wider social experiences (Colvert et al. 2008; Fries and Pollak 2004; Hwa‐Froelich et al. 2017; Kreppner et al. 1999; Malcorps et al. 2023; Tarullo et al. 2016; Vorria et al. 2006; Zournatzidis et al. 2025).

Preliminary evidence shows that some adult adoptees with early institutional deprivation significantly underperform on emotion recognition tasks compared to adoptees with no early deprivation (Golm et al. 2021). Furthermore, adolescents with early institutional care may exhibit heightened mistrust and difficulties discerning the trustworthiness of others, especially when adoption occurs at older ages (Pitula et al. 2017). From a salutogenetic perspective, greater mistrust may have negative implications for mentalizing, as mistrust can limit the individual's capacity to benefit from interpersonal relationships (Luyten et al. 2020). This may be especially relevant during adolescence, a period characterized by increasing reliance on peer relationships as contexts for sharing, reflecting on, and revising one's understanding of mental states.

Evidence from nonadopted samples supports the importance of early attachment in socio‐cognitive development. Meta‐analytic findings indicate that attachment security in childhood is associated with better emotion understanding, false‐belief understanding, and theory of mind (Szpak and Białecka‐Pikul 2020; Zeegers et al. 2019). However, the extent to which these associations persist across longer developmental periods remains unclear. Long‐term longitudinal studies examining associations between early attachment and later mentalizing are limited, yield mixed findings, and vary in their assessments of attachment (Sirparanta et al. 2025; Steele et al. 2016; Steele and Steele 2022). This highlights the need for more long‐term longitudinal research using standardized measures of attachment and mentalizing, particularly in samples exposed to early adversity.

### 1.1 The Present Study

The Greek Metera Study is a longitudinal investigation that has followed adopted and nonadopted individuals for more than two decades. At baseline, infants aged 11–17 months residing at the Metera Babies Centre were compared to infants reared in their biological families and attending a full‐time day‐care center (T1; Vorria et al. 2003). In the institution, each pavilion was staffed by 12 caregivers for 12 infants. However, the effective infant—caregiver ratio ranged from 4:1 to 6:1 because of the caregivers' 24‐h shifts. Furthermore, the mean overall quality of care at the institution (e.g., personal care, language experiences, creativity, and social experiences) was comparable to the daycare centers attended by the comparison infants. However, in terms of sensitivity, although staff interacted with the infants in care as frequently as the biological mothers in the comparison group, the quality and appropriateness of their interactions were lower in the institution (for a detailed description see Vorria et al. 2003).

**FIGURE 1:**
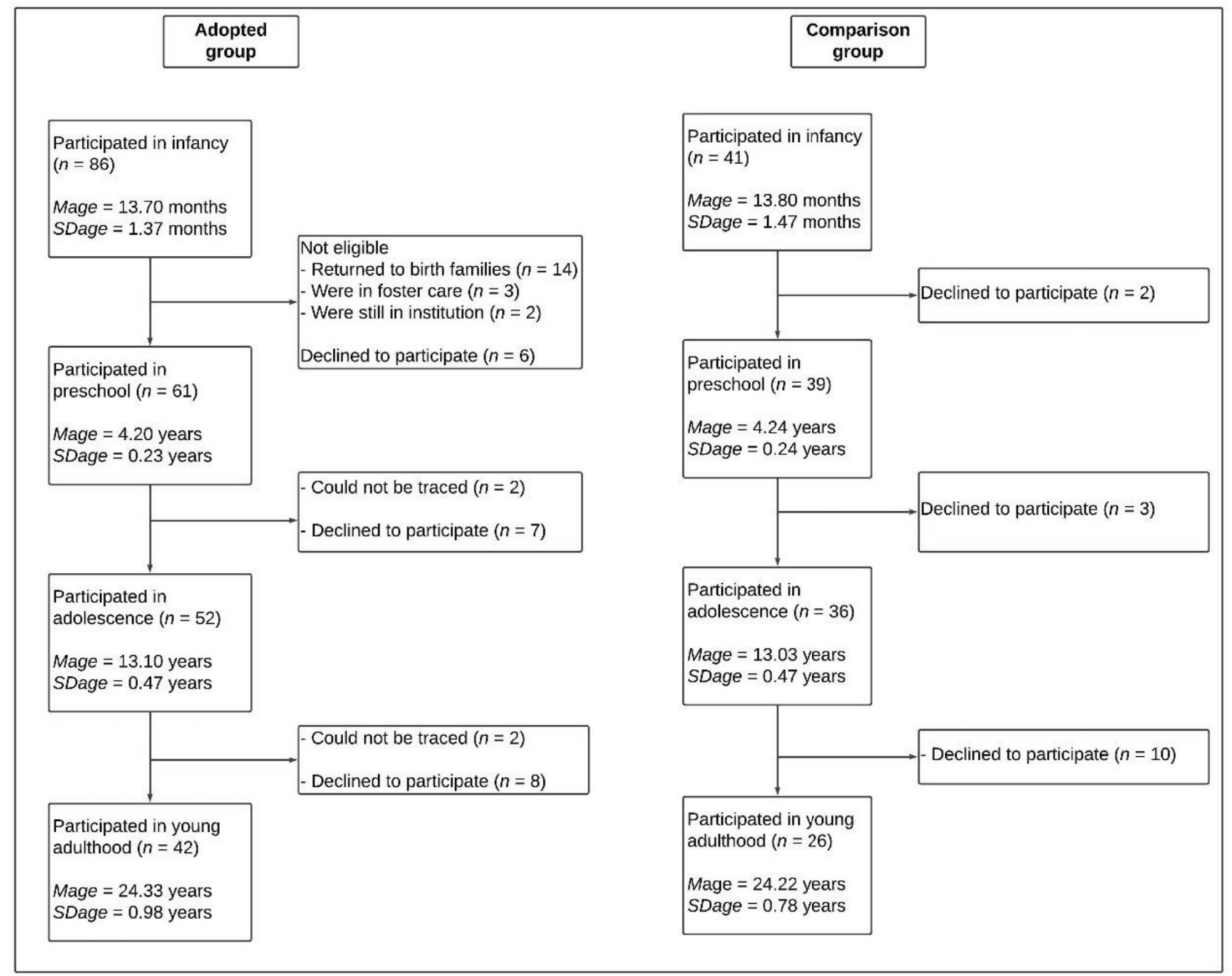
Participants flowchart and mean age in the Metera Study.

The infants from the institution were adopted and both groups were subsequently followed in preschool (T2; Vorria et al. 2006), and in adolescence (T3; Vorria et al. 2015). The present study is the adulthood follow‐up of the Metera Study (T4). A flowchart detailing participation across all waves is presented in Figure 1. Findings from the preschool follow‐up indicated that adopted children underperformed in emotional understanding compared to nonadopted peers (Vorria et al. 2006).

Considering the potential role of early sensitive periods in the development of mentalizing, together with the ongoing importance of social learning processes (Luyten et al. 2020), the present study examines whether early institutional care and the quality of attachment across childhood and adolescence are associated with RF in adulthood. In addition, it investigates whether trust in friendships, as a key context for sharing and reflecting on mental states during adolescence, contributes to individual differences in mentalizing in adulthood.

#### 1.1.1 Hypotheses

*Group status will moderate RF in adulthood, with late adoptees showing significantly lower scores on RF than the comparison group.*

*Later age at adoption and longer duration of institutional care will be negatively associated with RF in the adoption group.*

*Attachment insecurity in adolescence will predict lower RF in adulthood.*

*Quality of friendships at age 13 will positively be associated with RF at age 24.*

*Higher mistrust in friendships during adolescence will predict lower RF in adulthood.*

*In separate models, it was expected that mistrust in friendships at age 24, coherence of transcript from the Adult Attachment Interview (AAI), and dismissing and preoccupied states of mind at age 24, would mediate the association between attachment insecurity in adolescence and lower RF in adulthood.*

Further exploratory analyses were preregistered to explore (1) sex differences in RF; (2) the association between attachment in infancy and preschool with RF in adulthood.

## 2 Methods

### 2.1 Participants

Forty‐two adoptees (M_age_ = 24.33 years, SD = 0.98, range = 22.75–26.17) participated in the present follow‐up study, including 21 (50%) women and 21 men. The median age of admission to institution was 33 days (range = 4–344). Of the 42 adoptees, 36 (86%) were admitted before 3 months of age. Twenty (47.6%) adoptees were admitted to the institution because their mothers faced serious socioeconomic difficulties. The remaining 22 (52.4%) comprised a heterogeneous group of children with a history of maternal intellectual or physical disability, mental disorder, or drug addiction. Children were adopted at a mean age of 22.01 months (S*D* = 7.92; range = 12–38.80). The comparison group consisted of 26 nonadoptees brought up in their biological families (14 women, 53.8%; 12 men, 46.2%), and at the time of assessment their mean age was 24.22 years (SD = 0.78, range = 22.67–26.25).

Of the initial cohort of 86 infants in institutional care, 19 (22%) were not adopted and were not followed up. Of the remaining 67 adoptees, 42 (63%) participated in T4. In the comparison group, 26 out of the initial 41 (63%) participated in adulthood. In total, 68 (63%) out of the 108 individuals eligible for the adult phase of the study were successfully followed up. Attrition analyses were conducted as preregistered showing negligible selective attrition (see Supplemental Materials Table S1).

### 2.2 Measures

#### 2.2.1 Attachment (Age 1, 4, 13, and 24)

Attachment in infancy (Vorria et al. 2003) was measured with the strange situation procedure (SSP; Ainsworth et al. 1978), in preschool (Vorria et al. 2006) both with the attachment story completion task (ASCT; Bretherton et al. 1990) and the observer version of AQS (Waters and Deane 1985), and in adolescence (Vorria et al. 2015) with the child attachment interview (CAI; Target et al. 2003). Adult attachment representations were assessed using the adult attachment interview (AAI; George et al. 1985/1996). The AAI was explored as a categorical variable using the 2‐way classification system (secure vs. insecure). The coherence of transcript scale (CoT) as well as dismissing and preoccupied indices were used for dimensional analyses. The dismissing and preoccupied factors were computed in line with prior psychometric studies (Raby et al. 2017; Raby et al. 2022). Specifically, the dismissing factor (Ds) was calculated by averaging the idealization (toward both mother and father) and lack of recall scales. The preoccupied factor (E) was derived from averaging anger (toward both mother and father), passivity of thought, and unresolved scales (for past trauma and loss). All AAIs were rated by the first author. A second certified coder independently rated 25% of transcripts, yielding perfect agreement for secure versus insecure and unresolved classifications, and excellent agreement for insecure subclassifications, κ = 0.90 (see Zournatzidis et al. 2026; for details).

#### 2.2.2 Reflective Functioning (Age 24)

The Reflective Functioning Scale (RFS; Fonagy et al. 1998) was applied to the AAI to assess mentalizing. Scores range from −1 to 9, with values < 3 indicating poor mentalizing. Scores between 3 and 5 reflect average mentalizing, and scores > 5 suggest sophisticated RF. Regarding its latent structure, Taubner et al. (2013) reported that both one‐ and two‐factor solutions fit well (distinguishing RF in past vs. current relationships), although the highly correlated factors favor a more parsimonious one‐factor model. The first author rated all transcripts, and the third author independently rated 37 of the 68 transcripts (54%). Both coders were trained and certified at the Anna Freud Centre. Inter‐rater reliability was acceptable for individual ratings (ICC = 0.78, *p* < 0.001) and very high for mean RF scores (ICC = 0.88, *p* < 0.001).

#### 2.2.3 Age at Adoption

As preregistered, two cutoff points were used to distinguish early and late adoptees: Children adopted at or before 18 months (EA18) or 24 months (EA24) were classified as early adoptees, whereas those adopted after 18 or 24 months were classified as late adoptees (LA18 and LA24, respectively).

#### 2.2.4 Friendships (Age 13 and 24)

Peer relationships were assessed at age 13 using the Friendship Quality Questionnaire‐Revised (FQQ‐R; Parker and Asher 1989), and at age 24 using the McGill Friendship Questionnaire–Friendship Functions (MFQ‐FF; Mendelson and Aboud 1999). The intimate disclosure scale of the FQQ‐R and the intimacy scale of the MFQ‐FF were used to assess interpersonal trust in adolescence and adulthood respectively reflecting the extent to which individuals experience close friends as trustworthy (e.g., my friend “is someone I can tell private things to”). Both scales had Cronbach's *α* > 0.88 and the MFQ‐FF has shown good internal structure, reliability and validity both in Greek and non‐Greek adults (Mendelson and Aboud 1999; Pezirkianidis et al. 2022; Wagner 2019).

#### 2.2.5 Emotional Understanding (Age 4)

Children's emotional understanding was assessed using the Denham Puppet Scenario at age 4 (Denham 1986). This task used human‐like, but race and gender‐neutral puppets with interchangeable facial expressions (happiness, sadness, anger, and fear), and children were asked to choose the expression that matched the emotion in each scenario. Cronbach's *α* was acceptable (*α* = 0.77) (Vorria et al. 2006).

### 2.3 Procedure and Masking

Data were collected in person at participants' homes, with two assessments conducted online due to travel constraints. Participants from the adolescent wave were contacted by the PI, who knew the families from earlier follow‐ups, and appointments were scheduled at participants' convenience. During data collection, the authors who rated the transcripts were blind to participants' group status, unless this was disclosed spontaneously during the interview. To minimize bias when coding the AAI, the PI removed identifying information and assigned new pseudonymized IDs. A blindness score (0 = *completely blind*, 1 = *probably not blind*, 2 = *not blind*) was calculated to reflect the coder's awareness of group status. Blindness level was not significantly associated with AAI classifications. Participation was voluntary, with written informed consent obtained from young adults. Data collection began in 2020 and was extended because of COVID‐19 restrictions in Greece. Ethical approval for the study was granted by the Aristotle University of Thessaloniki.

### 2.4 Analysis Plan

As preregistered, one‐way ANOVAs examined differences between early adoptees, late adoptees, and the comparison group. As no significant group effects emerged, a nonpreregistered *t*‐test compared RF between the combined adopted group and the comparison group. Pearson correlations were used to test hypotheses [Link], [Link]. For exploratory analyses of attachment (secure vs. insecure) as a predictor of adult RF, ordinary least squares regression was used. Although regression analyses were preregistered to examine whether friendship variables predicted RF, these analyses were conducted using zero‐order correlations instead, as only one predictor was involved, and the results would be identical to those from regression. To investigate developmental pathways (H3a–H3d), mediation analyses with observed variables were performed using SEM with maximum likelihood estimation. Analyses were run on the total sample, followed by multigroup comparisons (adoptees vs. comparison group). Indirect effects were assessed using Sobel's test, supplemented by a nonpreregistered bootstrap procedure (5000 iterations) to estimate standard errors and test robustness of Sobel's result. As models were fully saturated, fit indices were uninformative, and path coefficients were not constrained to be equal across groups.

Power analyses conducted in our preregistration indicated that the present study was adequately powered to detect medium to large effects. Specifically, the study had sufficient power to detect a medium size for bivariate correlations (*r* = 0.34), a large between‐group difference for unequal group sizes (Cohen's *d* = 0.70), and a medium effect in regression with two predictors (*f* ^2^ = 0.15), with statistical power set at 0.80 and *α* = 0.05.

### 2.5 Transparency and Openness

We deviated from the preregistered plan in the following ways: (1) We explored whether disorganized attachment in infancy predicted RF in adulthood; (2) because RF scores were relatively low, exploratory analyses compared RF between secure and insecure AAI classifications in adulthood; (3) following a reviewer's recommendation, it was explored whether stability profiles of attachment and changes in interpersonal trust from adolescence to adulthood were associated with RF in adulthood.

## 3 Results

Descriptive statistics of RF at age 24 are presented in Table 1. Because RF scores were relatively low, exploratory (not preregistered) analyses compared RF between secure and insecure classifications. Those classified as secure showed substantially higher RF (M = 3.51) than those classified as insecure (M = 2.03), *t*(66) = 4.74, *p* < 0.001, Cohen's *d* = 1.15, 95% CI [0.63–1.67]. Although women tended to have higher mean RF scores (M = 3.11, SD = 1.60) compared to men (M = 2.45, SD = 1.28), this difference did not reach statistical significance *t*(66) = −1.87, *p* = 0.066, Cohen's *d* = 0.45, 95% CI [−0.94, 0.04].

**TABLE 1:** Descriptive statistics of reflective functioning at Age 24.

	*n*	M	SD	Min	Max
Total sample	68	2.79	1.48	0	7
Adopted	42	2.83	1.50	0	7
Comparison	26	2.73	1.48	1	6
Early adoptees (adopted ≤ 18 months)	14	2.93	1.38	1	5
Late adoptees (adopted > 18 months)	28	2.79	1.57	0	7
Early adoptees (adopted ≤ 24 months)	27	3.00	1.54	1	7
Late adoptees (adopted > 24 months)	15	2.53	1.41	0	5

Contrary to our expectations, group (comparison, early and late adopted) did not have a significant effect on RF in adulthood, regardless of whether the categorization of late adoptees was based on the 18‐month threshold, *F*(2, 65) = 0.08, *p* = 0.924, *η* ^2^ = 0.00, 95% CI [0.00, 1.00], or the 24‐month threshold, *F*(2, 65) = 0.51, *p* = 0.603, *η* ^2^ = 0.02, 95% CI [0.00, 1.00]. Correlations between age at admission in residential care, age at adoption, and duration in care with adult RF were all very small and nonsignificant (*r* = −0.09 to 0.04, all ns). Following a reviewer's suggestion, it was explored whether age at adoption and duration of institutional care interacted in predicting RF in adulthood. The interaction effect was not significant (*b* = 0.00, *p* = 0.594). Hence, subsequent analyses did not differentiate between early and late adoptees to increase statistical power. This decision was further supported by exploratory sensitivity analyses, which indicated no significant differences between early and late adoptees across key variables.

Zero‐order correlations between key variables are presented in Table 2 for the total sample. From a dimensional perspective, neither preschool ASCT coherence nor coherence in the CAI during adolescence significantly correlated with RF in adulthood. A significant negative correlation was found only between preschool attachment, as measured by the AQS, and adult RF (*r* = −0.27, *p* = 0.028). Categorical analyses indicated that attachment classifications assessed at earlier developmental stages did not significantly predict RF in adulthood. In infancy, regression analyses showed no significant association between attachment classification—whether secure versus insecure or organized versus disorganized—and RF (*b* = −0.21, *p* = 0.633; *b* = −0.22, *p* = 0.591, respectively). Similarly, attachment classification in preschool and adolescence did not predict RF in adulthood (*b* = 0.46 and *b* = 0.21 both ns). Contrary to hypotheses, trust in friendships during adolescence or adulthood was not associated with RF (see Table 2), and none of the adolescent friendship dimensions correlated with RF in adulthood, with Pearson correlations ranging from −0.06 to 0.08 (see Supplementary Material Table S2).

**TABLE 2:** Zero order correlations among key variables in the total sample (years).

Variable	RF
Coherence of transcript AAI (24)	0.49***
Dismissing factor AAI (24)	−0.58***
Preoccupied factor AAI (24)	0.29*
Trust in friends (24)	0.07
Trust in friends (13)	0.05
Quality of friendships (13)	0.02
Coherence CAI (13)	0.05
ASCT coherence (4)	0.04
AQS security (4)	−0.27*
Emotion understanding (4)	0.12

Abbreviations: AAI = adult attachment interview, AQS = Attachment Q‐Sort, ASCT = attachment story completion task, CAI = child attachment interview, RF = reflective functioning.

* *p* < 0.05.

** *p* < 0.01.

*** *p* < 0.001.

In terms of developmental pathways linking attachment insecurity in adolescence to lower RF in adulthood, indirect effects through adult mistrust in friendships or adult attachment dimensional indices (CoT, Ds, and E factors) were nonsignificant and ranged from −0.09 to 0.10. Path coefficients are reported in the Supplementary Material Figures S1–S8.

**FIGURE 2:**
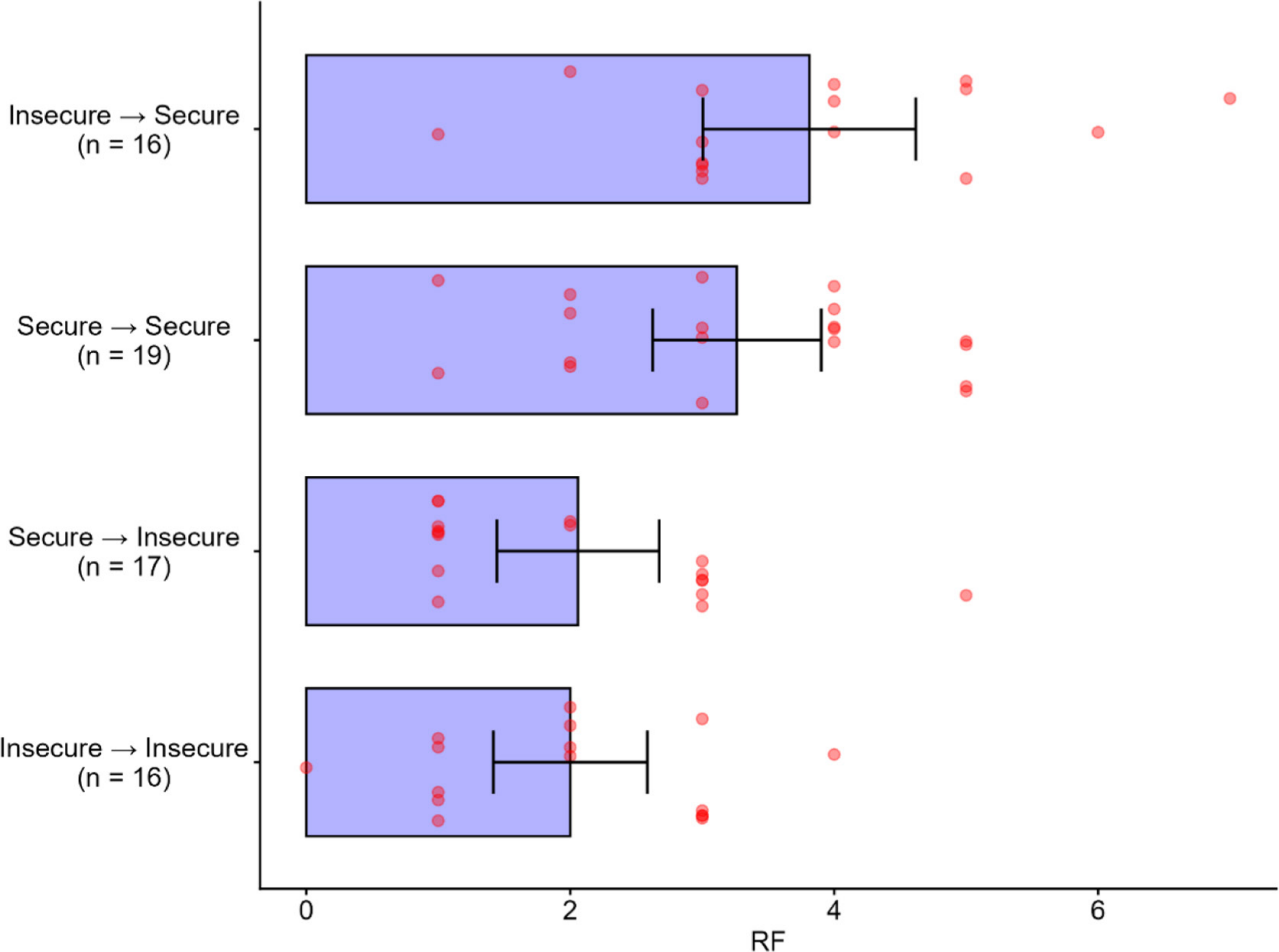
RF scores by attachment stability profiles from adolescence to adulthood. *Note:* Bars represent group means of RF, with error bars indicating 95% confidence intervals.

Following a reviewer's suggestion, exploratory analyses were conducted examining whether developmental trajectories of attachment and interpersonal trust from adolescence to adulthood were associated with adult RF. Attachment stability profiles from adolescence to adulthood (secure → secure; secure → insecure; insecure → secure; insecure → insecure) were examined. Descriptive statistics and pairwise comparisons are reported in the Supplementary Material Tables S3 and S4. The patterns observed suggest that differences were primarily driven by attachment classification in adulthood rather than by stability profiles (see Figure 2). Finally, interpersonal trust showed moderate stability from adolescence to adulthood (*r* = 0.32, *p* = 0.007). To examine whether developmental changes in trust were associated with adult RF, both measures were standardized and a change score was computed reflecting the relative change of trust from adolescence to adulthood. Changes in trust were not significantly associated with RF at age 24 (*r* = 0.07, *p* = 0.576), suggesting that increases or decreases in interpersonal trust did not predict RF in adulthood. These analyses should be interpreted cautiously, given the sample size and the limited power to detect trajectory‐based effects.

## 4 Discussion

The present study aimed to examine socio‐cognitive catch‐up among adult adoptees who experienced institutional care in infancy and explore predictors of RF in adulthood. Regarding the first aim, it was hypothesized that adoptees would show lower RF than the comparison group in adulthood, and that age at adoption would negatively affect RF. Contrary to expectations, RF at age 24 did not differ between groups, and age at adoption was not correlated with RF. These findings are consistent with the possibility of developmental plasticity in socio‐cognitive functioning. However, it is worth noting that RF was relatively low in the present sample. Community samples assessed with the Reflective Functioning Scale typically average around 3 and above (Fonagy et al. 1998; Jessee et al. 2016; Taubner et al. 2013), while the mean RF score in the present sample was 2.79. This could be attributable to the AAI context in which RF was assessed, while cultural factors may also play a role. Specifically, the majority (82%) of insecure classifications were dismissing, which is characterized by discourse‐limiting strategies and the mean RF score of those classified as secure was comparable to that reported in other nonclinical studies. Moreover, the very good interrater reliability of the RF ratings, together with the significant correlations in expected direction between RF and adult attachment dimensions, support the reliability and validity of the RF measure in this study.

As expected, the AAI was positively associated with RF cross‐sectionally. Significant overlap has been observed in other studies between adult attachment representations and RF (Fonagy et al. 1998; Taubner et al. 2013). Although this may be partly attributable to shared method variance, psychometric studies indicate that RF is not reducible to AAI classification (Jessee et al. 2016). However, it remains unclear whether RF, as coded from AAI transcripts, reflects representations of attachment experiences, or whether the AAI itself may be tapping into broader mentalizing capacities. Hence, the observed association should be interpreted with caution as it may reflect, at least in part, current attachment‐related processes, narrative coherence, or interview performance rather than a purely cumulative socio‐cognitive capacity. Moreover, AAI‐based RF may be relatively insensitive to early‐emerging mentalizing difficulties that were later compensated for through subsequent relational and developmental experiences, suggesting that the present null findings are theoretically informative and raise important questions about what AAI‐based RF captures developmentally. Finally, it should also be noted that the use of the same raters to score both RF and the AAI may have artificially inflated the observed associations.

In terms of longitudinal associations between early attachment and adult RF, the present results partly contrast with those of Sirparanta et al. (2025), who found that a composite attachment measure at age four predicted RF at age 23. As noted by the authors, the composite score should be interpreted with some caution in terms of its psychometric properties as an attachment measure. The present study drew on well‐validated attachment measures across development (i.e., strange situation, attachment story completion task, CAI) and found a consistent pattern of nonsignificant longitudinal associations between early attachment and adult RF. Although AQS at age 4 was correlated with RF in adulthood, the direction of the association was opposite to that reported by Sirparanta et al. (2025), suggesting that attachment insecurity in preschool, as assessed behaviorally by the AQS, was associated with higher RF in adulthood. This finding was counterintuitive and warrants cautious interpretation, particularly as it runs counter to the pattern observed from all other attachment measures in the present study (SSP, ASCT, and CAI). Given the heterogeneity in measures of early attachment and the mixed findings from previous studies (Sirparanta et al. 2025; Steele et al. 2016; Steele and Steele 2022), further research is still needed in larger studies to replicate and further explore longitudinal association between attachment in childhood and RF in adulthood.

Although the predictive power of early attachment for socio‐cognitive outcomes may diminish over longer developmental periods, its association with RF in adulthood is likely to be nonlinear. This is consistent with evidence indicating modest stability of attachment from infancy to adulthood (Pinquart et al. 2013), a pattern also observed in the present sample (Zournatzidis et al. 2026). In parallel, RF itself may be sensitive to change, fluctuating over time in response to ongoing contextual influences (Fiore et al. 2026). In exploring potential mediation pathways, the association between attachment insecurity in adolescence and RF in adulthood was not mediated by interpersonal trust or adult attachment representations. These findings may suggest that the pathways linking attachment to adult mentalizing are more complex than initially assumed and may involve additional contextual and relational processes not captured in the present study. Developmental transitions from adolescence to adulthood such as leaving the parental home, entering higher education or employment, and forming romantic relationships may contribute to this reorganization over time.

Contrary to our hypotheses, mistrust in friendships in both adolescence and adulthood was not associated with RF in adulthood. Most individuals demonstrated high levels of interpersonal trust which may have reduced the variability needed to detect associations with RF. Furthermore, while interpersonal trust reflects a general willingness to rely on others, it does not capture how individuals respond to information shared by others or whether their trust reflects an adaptive epistemic stance. High interpersonal trust may not indicate whether trust is selective and discerning, or whether individuals are vulnerable to credulity—patterns more commonly observed in individuals with mental health difficulties (Li et al. 2023). Future studies are needed to directly assess epistemic trust and examine whether it prospectively predicts RF across development.

### 4.1 Strengths and Limitations

A major strength of this study is its longitudinal design, spanning more than two decades with repeated, well‐validated attachment assessments from infancy to young adulthood. Although attrition rates were modest, 22% of those lost at the preschool wave were excluded because they had not been adopted. Additionally, the sample is relatively homogeneous in terms of early adversity, as early institutional care was not compounded by major preadoption risks (e.g., maltreatment), allowing to explore the effects of institutional care without other significant confounds. Finally, preregistration of hypotheses and analytic plan increased the transparency and methodological rigor of the study.

Despite its strengths, the study also has limitations. First, although the sample size was reasonable for a long‐term project using labor‐intensive measures (e.g., CAI, AAI, and RFS) and was adequately powered to detect medium to large effects, it remained insufficient to detect small to medium effects. Second, the sample largely comprised low‐risk families, which may limit generalizability to populations experiencing greater socioeconomic or psychosocial adversity. Finally, interpersonal trust within friendships may not fully capture epistemic mistrust or epistemic credulity, which are more directly linked to RF.

### 4.2 Future Research

Future studies should further examine the developmental pathways of RF in adulthood by considering the roles of attachment, epistemic trust, and broader environmental influences. Given the strong association between RF and attachment when both are assessed via the AAI, it will be important to assess RF in contexts beyond attachment‐related interviews, but also using performance‐based or nonverbal assessments of mentalizing. Research should also address the stability of RF and investigate the influence of wider social relationships, including mentors, educators, and romantic partners. Finally, future work should explore the role of parental reflective functioning during the transition from adolescence to adulthood.

### 4.3 Conclusions

Despite earlier difficulties in emotional understanding at age four, adoptees with early institutional experience did not differ from their nonadopted peers in RF in adulthood, highlighting the potential positive influence of adoptive families on socio‐cognitive recovery. Although RF was strongly associated with adult attachment representations cross‐sectionally, further longitudinal research is needed to better understand the developmental pathways of RF into adulthood, particularly the role of early attachment, epistemic trust, and later social experiences.

## Funding

This work was supported by the International Psychoanalytical Association (RG2326).

## Conflicts of Interest

The authors declare no conflicts of interest.

## Supporting information


**Table S1:** Attrition analyses.
**Table S2:** Correlations between friendship dimensions in adolescence and RF in adulthood in the total sample (years).
**Table S3:** Descriptive statistics for reflective functioning by attachment stability profiles.
**Table S4:** Pairwise comparisons for RF by attachment stability profiles from adolescence to adulthood.
**Figure S1:** Mediation by interpersonal mistrust in adulthood—total sample.
**Figure S2.** Mediation by interpersonal mistrust in adulthood—multigroup comparison. CAI = child attachment interview, RF = reflective functioning. Coefficients are standardized betas. *p < 0.05, **p < 0.01, ***p < 0.001.
**Figure S3.** Mediation by attachment in adulthood—total sample. AAI = adult attachment interview, CAI = child attachment interview, CoT = coherence of transcript; RF = reflective functioning. Coefficients are standardized betas. *p < 0.05, **p < 0.01, ***p < 0.001.
**Figure S4.** Mediation by attachment in adulthood—multigroup comparison. AAI = adult attachment interview, CAI = child attachment interview; CoT = coherence of transcript; RF = reflective functioning. Coefficients are standardized betas. *p < 0.05, **p < 0.01, ***p < 0.001.
**Figure S5.** Mediation by dismissing attachment in adulthood—total sample. AAI = adult attachment interview, CAI = child attachment interview, Ds = dismissing, RF = reflective functioning. Coefficients are standardized betas. *p < 0.05, **p < 0.01, ***p < 0.001.
**Figure S6.** Mediation by dismissing attachment in adulthood—multigroup comparison. AAI = adult attachment interview, CAI = child attachment interview, Ds = dismissing, RF = reflective functioning. Coefficients are standardized betas. *p < 0.05, **p < 0.01, ***p < 0.001.
**Figure S7.** Mediation by preoccupied attachment in adulthood—total sample*.* AAI = adult attachment interview, CAI = child attachment interview, E = preoccupied, RF = reflective functioning. Coefficients are standardized betas. *p < 0.05, **p < 0.01, ***p < 0.001.
**Figure S8.** Mediation by preoccupied attachment in adulthood—multigroup comparisons. AAI = adult attachment interview, CAI = child attachment interview, E = preoccupied, RF = reflective functioning. Coefficients are standardized betas. *p < 0.05, **p < 0.01, ***p < 0.001.

## Data Availability

The data that support the findings of this study are available on request from the corresponding author. The data are not publicly available due to privacy or ethical restrictions.
