# Supplementary material for: Mentalizing and Attachment From Infancy to Young Adulthood: Insights From the Metera Adoption Study
Source: Personal Ment Health. 2026 Jun 9;20(3):e70084. doi: 10.1002/pmh.70084 (PMC13248948; doi:10.1002/pmh.70084)
Supplement: Supplementary file 1 — Table S1: Attrition analyses. Table S2: Correlations between friendship dimensions in adolescence and RF in adulthood in the total sample (years). Table S3: Descriptive statistics for reflective functioning by attachment stability profiles. Table S4: Pairwise comparisons for RF by attachment stability profiles from adolescence to adulthood. Figure S1: Mediation by interpersonal mistrust in adulthood—total sample. Figure S2. Mediation by interpersonal mistrust in adulthood—multigroup comparison. CAI = child attachment interview, RF = reflective functioning. Coefficients are standardized betas. *p < 0.05, **p < 0.01, ***p < 0.001. Figure S3. Mediation by attachment in adulthood—total sample. AAI = adult attachment interview, CAI = child attachment interview, CoT = coherence of transcript; RF = reflective functioning. Coefficients are standardized betas. *p < 0.05, **p < 0.01, ***p < 0.001. Figure S4. Mediation by attachment in adulthood—multigroup comparison. AAI = adult attachment interview, CAI = child attachment interview; CoT = coherence of transcript; RF = reflective functioning. Coefficients are standardized betas. *p < 0.05, **p < 0.01, ***p < 0.001. Figure S5. Mediation by dismissing attachment in adulthood—total sample. AAI = adult attachment interview, CAI = child attachment interview, Ds = dismissing, RF = reflective functioning. Coefficients are standardized betas. *p < 0.05, **p < 0.01, ***p < 0.001. Figure S6. Mediation by dismissing attachment in adulthood—multigroup comparison. AAI = adult attachment interview, CAI = child attachment interview, Ds = dismissing, RF = reflective functioning. Coefficients are standardized betas. *p < 0.05, **p < 0.01, ***p < 0.001. Figure S7. Mediation by preoccupied attachment in adulthood—total sample. AAI = adult attachment interview, CAI = child attachment interview, E = preoccupied, RF = reflective functioning. Coefficients are standardized betas. *p < 0.05, **p < 0.01, ***p < 0.001. Figure S8. Mediation by preoccupi [file PMH-20-0-s001.docx]

**Supplementary** **materials**

**Table S1**

*Attrition Analyses*

|  | Continued ^a^ | Dropped out ^a^ | *p* value |
| --- | --- | --- | --- |
| Sex T1 |  |  | .934 |
| Male | 33 (49%) | 30 (51%) |  |
| Female | 35 (51%) | 29 (49%) |  |
| Age T1 (months) | 13.82 (1.61) | 13.73 (1.13) | .707 |
| Group |  |  | .177 |
| In care | 42 (62%) | 44 (75%) |  |
| Comparison | 26 (38%) | 15 (25%) |  |
| Attachment T1 |  |  | .455 |
| Secure | 19 (33%) | 13 (25%) |  |
| Insecure | 39 (67%) | 40 (75%) |  |
| Bayley Mental T1 | 96.30 (11.37) | 95.43 (9.74) | .648 |
| Bayley Motor T1 | 105.63 (16.79) | 98.87 (16.52) | .028 |
| Emotion understanding T2 | 21.00 (5.03) | 20.87 (6.93) | .926 |
| SDQ PA T3 | 8.31 (6.72) | 6.90 (5.50) | .346 |
| SDQ TEA T3 | 7.45 (7.20) | 7.75 (6.44) | .889 |
| SDQ CH T3 | 10.06 (4.54) | 8.40 (5.36) | .219 |
| Attachment T3 |  |  | .485 |
| Secure | 36 (53%) | 13 (65%) |  |
| Insecure | 32 (47%) | 7 (35%) |  |
| Social class T3 |  |  | .416 |
| Low | 20 (29%) | 3 (15%) |  |
| Middle | 41 (60%) | 15 (75%) |  |
| High | 7 (10%) | 2 (10%) |  |
| Education M years T3 | 12.55 (3.54) | 12.30 (3.25) | .767 |
| Education F years T3 | 12.36 (3.52) | 11.50 (3.24) | .313 |

*Note.* ^a^ = n (%); Mean (SD). T1 = infancy; T2 = preschool; T3 = adolescence; SDQ = Strengths and Difficulties Questionnaire; PA = parent; TEA = teacher; CH = child; M = mother; F = father

**Table S2**

*Correlations Between Friendship Dimensions in Adolescence and RF in Adulthood in the Total Sample* *(Years)*

| Variable | 1 | 2 | 3 | 4 | 5 | 6 | 7 |
| --- | --- | --- | --- | --- | --- | --- | --- |
| 1. RF (24) | — |  |  |  |  |  |  |
| 2. Companionship (13) | −.04 |  |  |  |  |  |  |
| 3. Validation & caring (13) | .02 | .42*** |  |  |  |  |  |
| 4. Guidance and help (13) | .08 | .62*** | .75*** |  |  |  |  |
| 5. Intimate disclosure (13) | .05 | .55*** | .79*** | .80*** |  |  |  |
| 6. Conflict betrayal (13) | −.06 | −.20 | −.37** | −.31* | −.40*** |  |  |
| 7. Conflict resolution (13) | −.02 | .42*** | .69*** | .72*** | .72*** | −.23 |  |

*Note.* RF = Reflective Functioning.

**p* < .05, ***p* < .01, ****p* < .001

**Table S3**

*Descriptive Statistics for Reflective Functioning by Attachment Stability Profiles*

|  | M | SD | Min | Max |
| --- | --- | --- | --- | --- |
| Secure → Secure (*n* = 19) | 3.26 | 1.33 | 1.00 | 5.00 |
| Secure → Insecure (*n* = 17) | 2.06 | 1.20 | 1.00 | 5.00 |
| Insecure → Secure (*n* = 16) | 3.81 | 1.52 | 1.00 | 7.00 |
| Insecure → Insecure (*n* = 16) | 2.00 | 1.10 | 0.00 | 4.00 |

**Table S4**
*Pairwise Comparisons for RF by attachment stability profiles from adolescence to adulthood*

| Comparison | *p* | Cohen’s *d* | 95% CI |
| --- | --- | --- | --- |
| Secure → Secure **vs** Secure → Insecure | .**034** | 0.93 | [0.26, 1.63] |
| Secure → Secure **vs** Insecure → Secure | .597 | −0.42 | [−1.14, 0.35] |
| Secure → Secure **vs** Insecure → Insecure | .**027** | 0.98 | [0.28, 1.63] |
| Secure → Insecure **vs** Insecure → Secure | .**001** | −1.36 | [−2.06, −0.54] |
| Secure → Insecure **vs** Insecure → Insecure | .999 | 0.05 | [−0.55, 0.66] |
| Insecure → Secure **vs** Insecure → Insecure | .**001** | 1.40 | [0.67, 2.08] |

*Note.* Tukey’s correction was applied to adjust for multiple comparisons.

**Figure S1**

*Mediation by Interpersonal Mistrust in Adulthood – Total Sample*


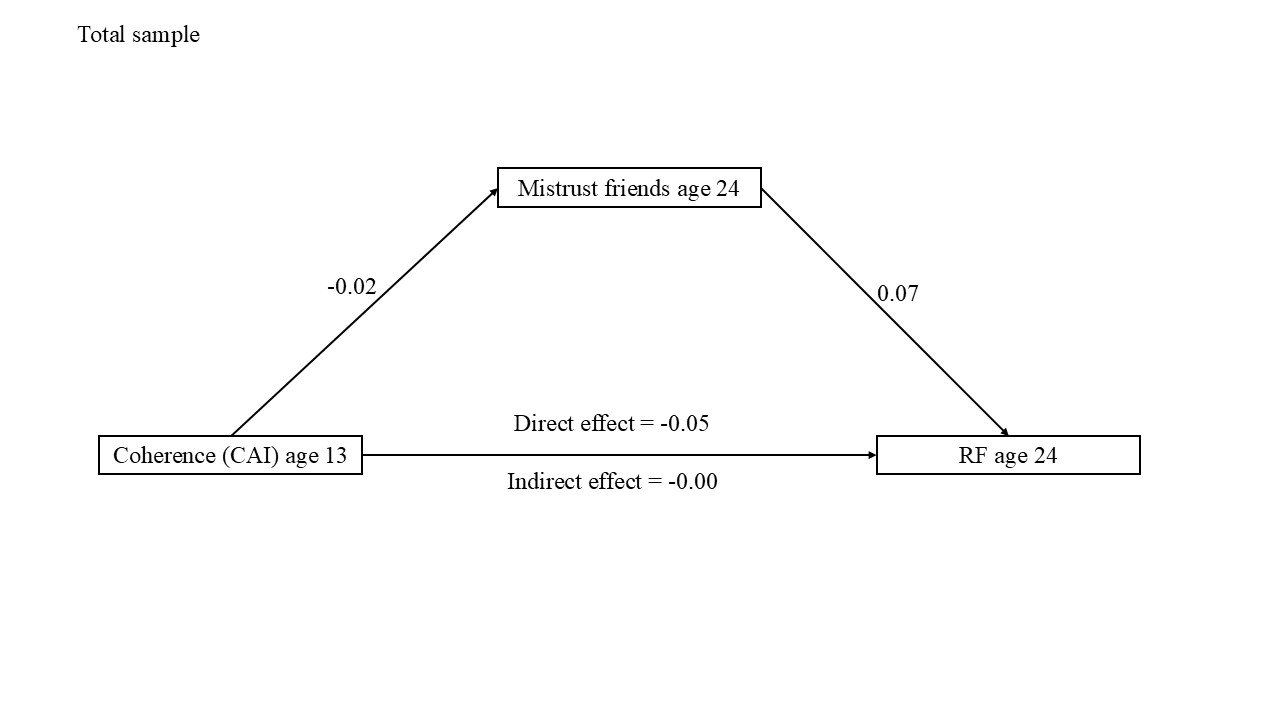


*Note.* CAI = Child Attachment Interview; RF = Reflective Functioning. Coefficients are standardized betas.

**p* < .05, ***p* < .01, ****p* < .001

**Figure 2**

*Mediation by Interpersonal Mistrust in Adulthood – Multigroup Comparison*


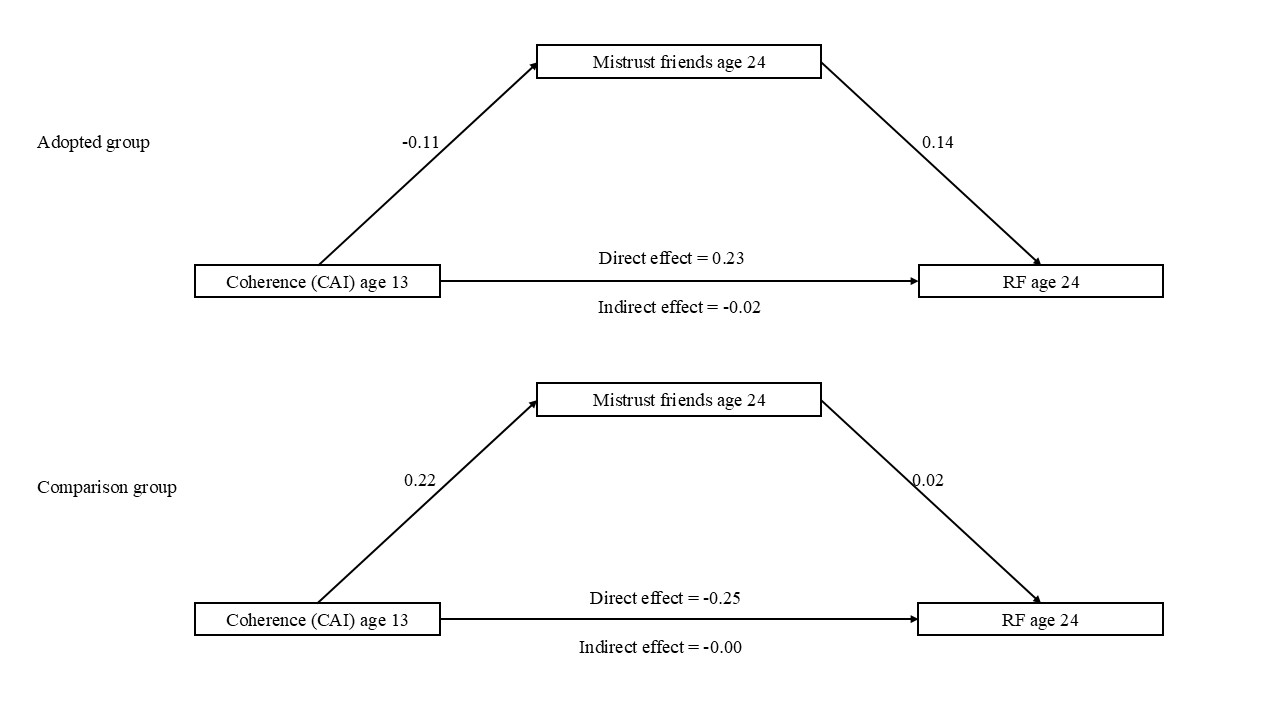


*Note.* CAI = Child Attachment Interview; RF = Reflective Functioning. Coefficients are standardized betas.

**p* < .05, ***p* < .01, ****p* < .001

**Figure 3**

*Mediation by Attachment in Adulthood – Total Sample*


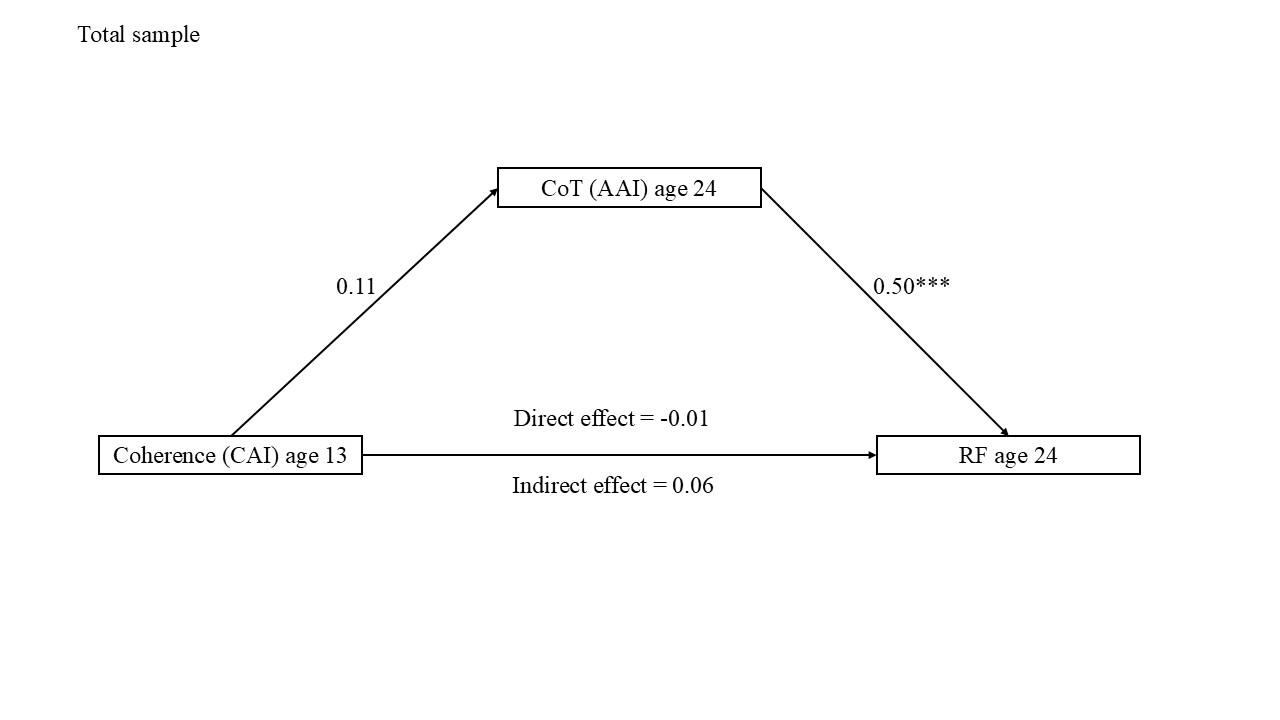


*Note.* CAI = Child Attachment Interview; CoT = Coherence of Transcript; AAI = Adult Attachment Interview; RF = Reflective Functioning. Coefficients are standardized betas.

**p* < .05, ***p* < .01, ****p* < .001

**Figure 4**

*Mediation by Attachment in Adulthood – Multigroup Comparison*


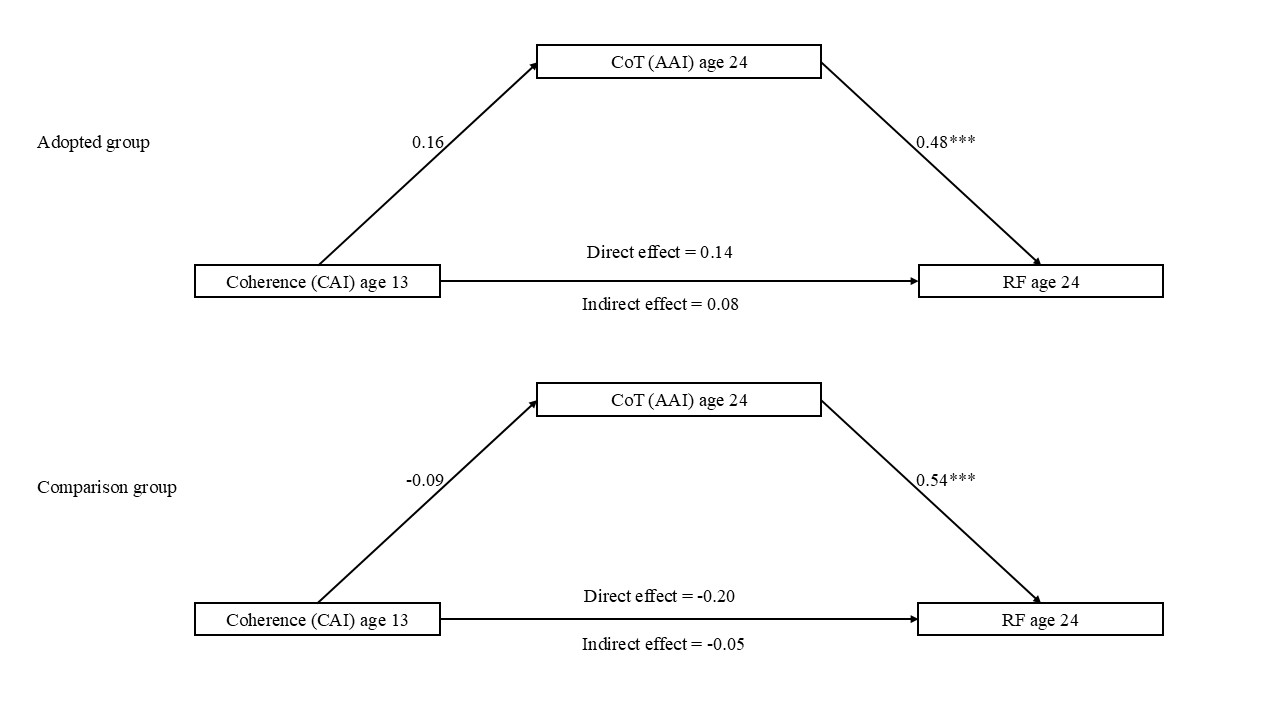


*Note.* CAI = Child Attachment Interview; CoT = Coherence of Transcript; AAI = Adult Attachment Interview; RF = Reflective Functioning. Coefficients are standardized betas.

**p* < .05, ***p* < .01, ****p* < .001

**Figure 5**

*Mediation by Dismissing Attachment in Adulthood – Total Sample*


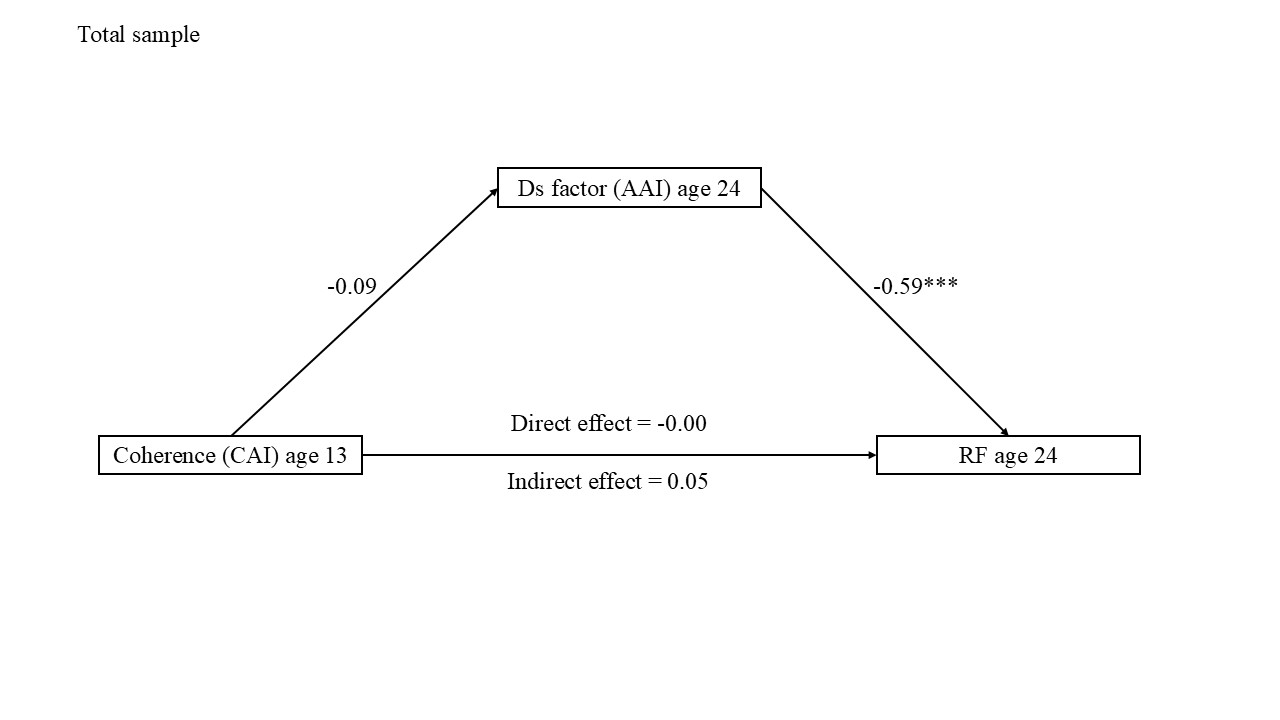


*Note.* CAI = Child Attachment Interview; Ds = dismissing; AAI = Adult Attachment Interview; RF = Reflective Functioning. Coefficients are standardized betas.

**p* < .05, ***p* < .01, ****p* < .001

**Figure 6**

*Mediation by Dismissing Attachment in Adulthood – Multigroup Comparison*


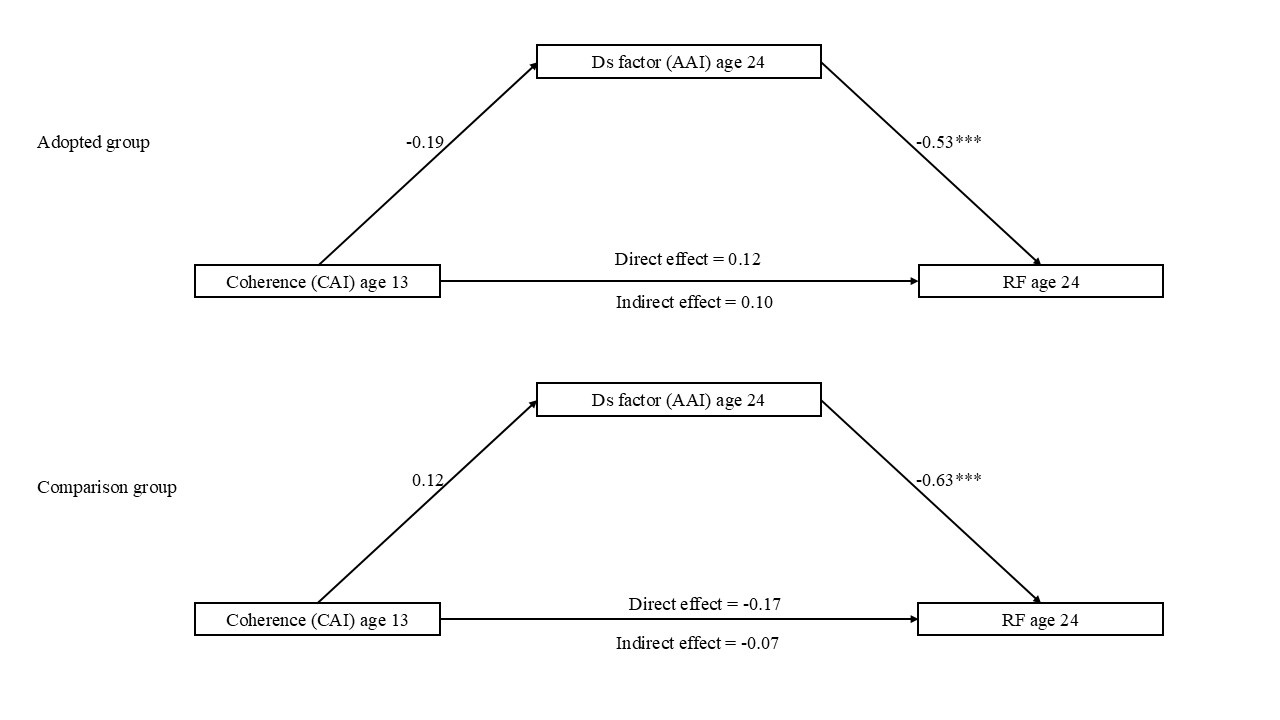


*Note.* CAI = Child Attachment Interview; Ds = dismissing; AAI = Adult Attachment Interview; RF = Reflective Functioning. Coefficients are standardized betas.

**p* < .05, ***p* < .01, ****p* < .001

**Figure 7**

*Mediation by Preoccupied Attachment in Adulthood – Total Sample*


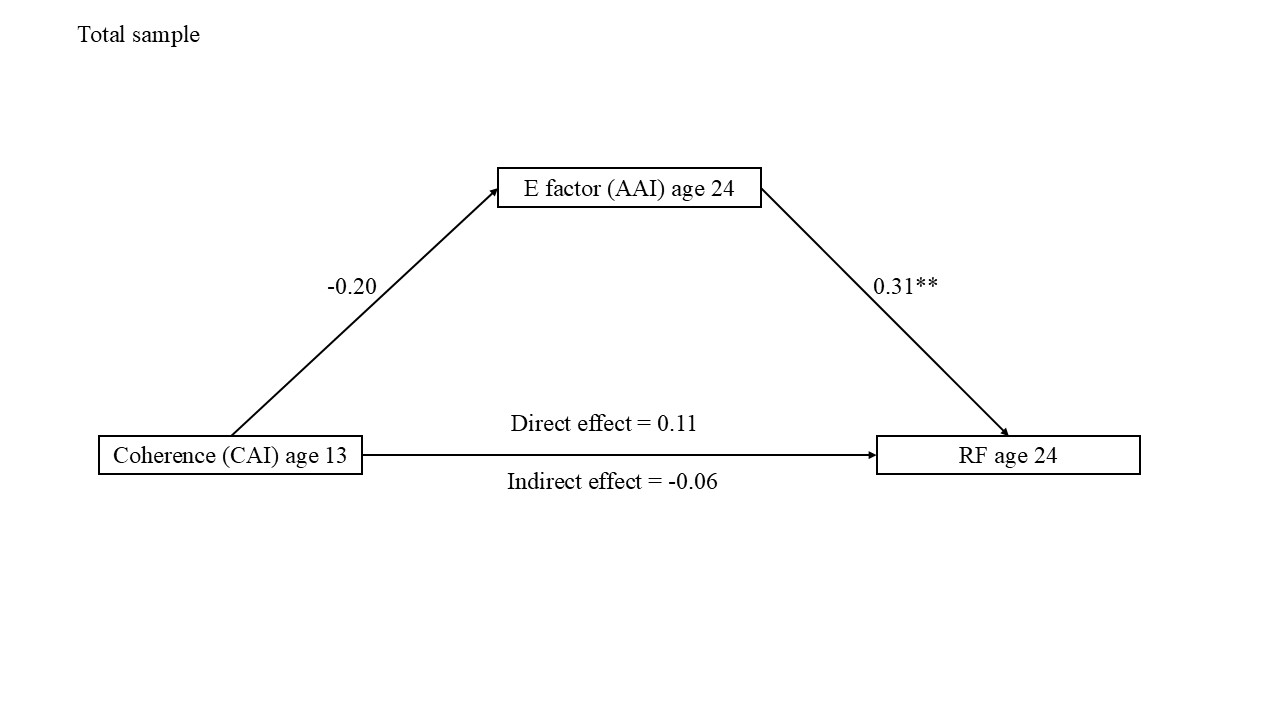


*Note.* CAI = Child Attachment Interview; E = preoccupied; AAI = Adult Attachment Interview; RF = Reflective Functioning. Coefficients are standardized betas.

**p* < .05, ***p* < .01, ****p* < .001

**Figure 8**

*Mediation by Preoccupied Attachment in Adulthood – Multigroup Comparisons*


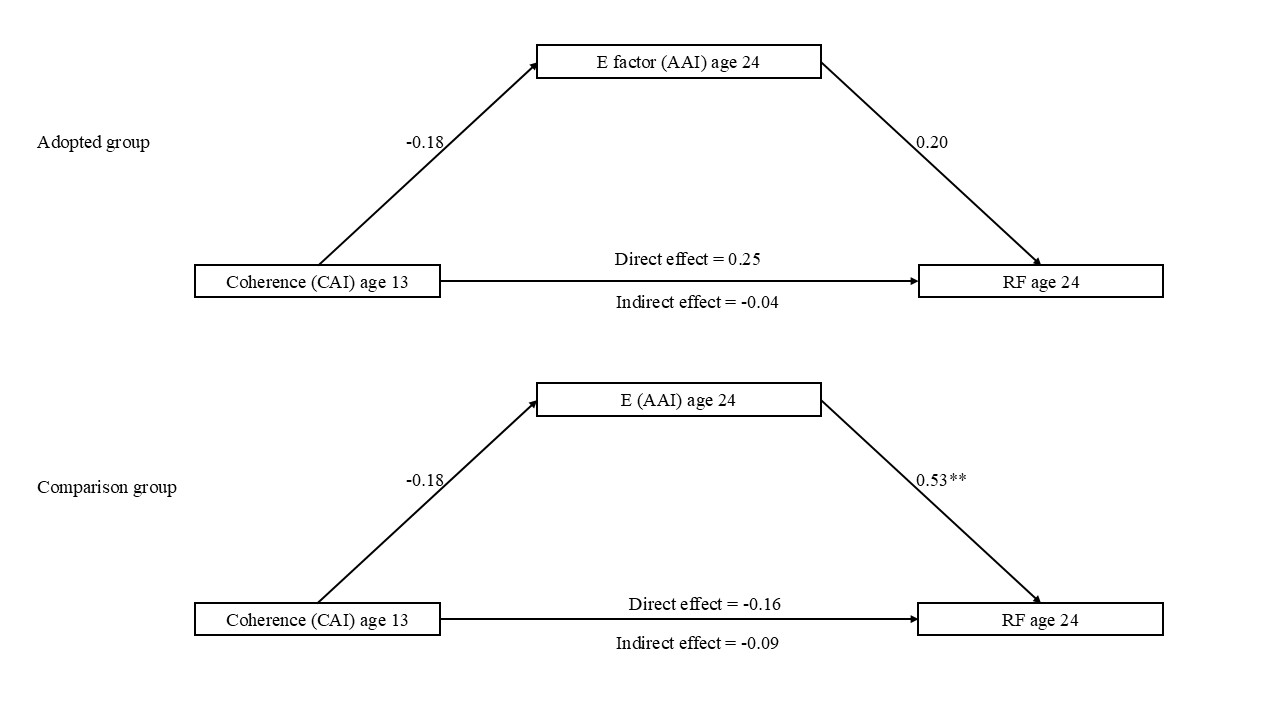


*Note.* CAI = Child Attachment Interview; E = preoccupied; AAI = Adult Attachment Interview; RF = Reflective Functioning. Coefficients are standardized betas.

**p* < .05, ***p* < .01, ****p* < .001
